# The Amendment of the Act

**Published:** 1913-04-12

**Authors:** 


### NATIONAL INSURANCE ACT DEPARTMENT.

### XLIX.—The Amendment of the Act.

Although there was no reference in the King's Speech on the opening of the new Session of Parliament to the amendment of the National Insurance Act, we now print indications pointing to the possibility of the early introduction of an amending Bill. The Act has been criticised perhaps more than any other measure in modern times, but whilst it is easy to point out its defects it is by no means so easy to suggest a way of amending them. Very wise statesmanship will be required in dealing practically with the matter to ensure that national interests shall not be subordinated to those of a sectional character. That this is so is evident from the remarks of a prominent official of one of the larger approved societies recently published in a journal circulating amongst the representatives of his society. After admitting that much of the work entailed by the Act in its present form is cumbrous and, as he thinks, unnecessary, he puts forward the suggestion whether it is not worth while to allow matters to remain as they are without pressing for amendment of any description. By so doing, it appears to him that the approved societies with the best organisation would stand to gain, because they will be able to stand the strain the longer. It need hardly be pointed out that if these better organised societies gain, it will be at the expense of the smaller and less well-organised societies, and it is therefore clear that jealousies between rival societies and organisations will have to be carefully guarded against. In the "scramble for members" which took place last summer, it has unfortunately to be admitted that the expression of these jealousies was only too common, but it was hoped that in course of time this unpleasant feature would have passed away, and it is somewhat disquieting to note its recurrence.

### A Comparison with Old-Age Pensions.

Now that the Act has been in operation for practically nine months, it is difficult to see how any root-and-branch amendment can be made. Having once been set in motion, such vast machinery as is here involved cannot well be put out of action.

Not only has National Insurance against sickness and unemployment come to stay, but the main lines of the system are permanently laid, and it is only in matters of detail that amendments will be feasible, unless, in the course of time, a Government should feel itself so strong as to be able to carry out the desire of the extreme section of the Socialists by the introduction of a non-contributory system such as that at present in vogue in regard to National Old Age Pensions. There does not appear at the moment to be any prospect of the success of any such far-reaching amendment of the system in the near future, either in this country or in any other, and it does not therefore call for further comment.

### The Scheme of the National Insurance Inquiry Association.

Of all the schemes for amendment which were at all comprehensive in character, the one put forward by the National Insurance Inquiry Association just twelve months ago was the most complete. It was published in the *Times* of April 4, 1912, and it may not be out of place briefly to consider the proposals then put forward, though it has to be admitted that as the Act has since come into operation some at least of the suggestions could not now be carried out practically. Under this scheme the contributions were to be the same as those laid down by the Act, but instead of the Government's contribution being paid as a proportion of the benefits and expenses as they emerge, it was to be paid as a proportion of the contributions themselves. The benefits, on the other hand, were to be considerably modified, and the scheme for their administration would have been radically altered. Instead of the present triple system of administration through Insurance Commissioners, Insurance Committees, and Approved Societies, the Inquiry Association recommended that there should be two distinct schemes.

The first and most important was the setting up of a Central Insurance Bureau with definitely guaranteed benefits open to all; and the second was to be of a supplementary character in the nature of subsidised society insurance which was to be administered on Friendly Society lines, with safeguards to ensure solvency. The benefits guaranteed under the Central Scheme would have included Maternity and Sanatorium Benefits almost exactly similar to those of the Act, and Disablement Benefit would have been extended by the application of twice as much provision as at present, so that payments could be made in cases of partial disablement when this course was shown to be desirable.

Medical and Sickness Benefits, which, under the Act absorb roughly two-thirds of the contributions, would, however, have been omitted, and in their place pensions of a minimum amount of 5s. a week, commencing at the age of sixty, and allowances to widows and orphans would have been substituted.

There is much that could be said in favour of these suggested alterations of benefits, but on the whole we prefer those of the Act, as they make provision for immediate payments which, although they may be less in actual amount, are more tangible than benefits, the payment of which is deferred to the age of sixty.

Guaranteed Benefits.

The most important point in our opinion is the suggestion of the guarantee of benefits. Under the Act the sickness and disablement benefits are set out in imposing array, but they are not guaranteed and, as we have previously pointed out, it may be necessary for some approved societies to reduce these benefits after the first valuation, whilst others will be in a position to increase them. This will not necessarily be the fault of the societies concerned, but will be due, apart from actual mismanagement, to the method of administration under which it was possible for large classes of the most healthy lives to form societies of their own. The contributions were based on actuarial tables applicable to insured persons as a whole, and there is no reasonable doubt that they are sufficient to provide the benefits when the law of averages is allowed full play. Although this may be so in regard to the scheme as a whole, it is well known that certain occupations conduce to a higher rate of sickness than others, and, if a society has drawn its members principally from one of these hazardous occupations, it can be foreseen with reasonable certainty that the contributions will be unequal to support the burden of the benefits laid upon the funds of the society.

In a truly National Scheme this anomaly should not exist. The fact of its existence is aggravated when it is remembered that the Government's contribution is applied as a proportion of the benefits, and therefore that those societies which grant the smallest rate of benefits will receive the smallest State subsidy. For the moment all societies are paying the same benefits, and the differentiation of rates of benefit as between different societies has not therefore arisen, and it cannot arise until the first valuations have been made. When, however, the results of the first three years' working have been tested by valuation, and the altered rates of benefits are brought into operation or, in the alternative, levies are made to liquidate the deficiencies, it is easy to imagine that dissatisfaction will be manifested.

We have already, in these columns, commented upon the unnecessary multiplication of authorities, as it appears to us, by the institution of the separate Commissioners for England, Scotland, Ireland, and Wales respectively. It is not only in regard to the increase of administrative costs and the trouble entailed by transfers that this division of authority is to be deprecated. Societies having members in more than one country of the Union are required to make separate valuations for the members in each of the countries, and the surplus, if any, disclosed, is to be retained for the benefit of the members in the respective countries. Thus, not only will rates of benefit vary as between different societies, but in the same society there may be different scales of benefits according to the country in which the member happens to reside.

The question is one which should undoubtedly receive careful attention, and the recommendation made by the Inquiry Association that the benefits should be guaranteed cannot be lightly thrown aside.

The Deposit Contributor Class.

It was thought that the deposit contributor class would be the most difficult to deal with, and provision was made in the Act that the present scheme in regard to them must be reviewed by Parliament before the end of next year. Curiously enough, it would seem that the one subject to obtain the distinction of special mention for review will have practically settled itself within the time allotted. There was, of course, no means of knowing how many persons would become deposit contributors, but an estimate was made on the assumption that they would be mostly unhealthy lives, whom the approved societies would not admit into membership. In point of fact, the deposit contributor class has fallen much below the expected estimate, and is of very different constitution, so far as can be gathered, from that anticipated. An almost insignificant number of insured persons have been rejected membership in approved societies on account of ill-health, and instead of being unhealthy lives, the deposit contributor class seems to consist very largely of those who found it suit their convenience to become Post Office contributors. Facilities for transfer of deposit contributors to approved societies have, very wisely, been granted beyond the limits laid down in the first regulations, and numerous transfers of this description have already taken place and are still occurring. The amendment of the present provisions of the Act in regard to deposit contributors will not, therefore, present the difficulties that were anticipated.

Medical Benefit and Hospital Treatment.

The amendment of the Act in regard to medical benefits has already received much attention in our columns. The regulations under which this benefit is now being administered are, at the best, only a temporary expedient, and the recognition of the services of the voluntary hospitals in the treatment of insured persons must be taken into account in the amendment of the Act on permanent lines.

---

NOTE.—Questions and Correspondence on all doubtful points are invited, and should be addressed to the Editor, The Hospital, 28, 29 Southampton Street, Strand, London, W.C., and marked "Insurance."

